# Inversion effects in the expert classification of mammograms and faces

**DOI:** 10.1186/s41235-018-0123-6

**Published:** 2018-08-15

**Authors:** Michael D. Chin, Karla K. Evans, Jeremy M. Wolfe, Jonathan Bowen, James W. Tanaka

**Affiliations:** 1 Department of Psychology, University of Victoria, Victoria, BC Canada; 2 University of York, York, UK; 3 Harvard Medical School, Boston, USA

**Keywords:** Holistic, Perceptual expertise, Radiology, Inversion

## Abstract

A hallmark of a perceptual expert is the ability to detect and categorize stimuli in their domain of expertise after brief exposure. For example, expert radiologists can differentiate between “abnormal” and “normal” mammograms after a 250 ms exposure. It has been speculated that rapid detection depends on a global analysis referred to as holistic perception. Holistic processing in radiology seems similar to holistic perception in which a stimulus like a face is perceived as an integrated whole, not in terms of its individual features. Holistic processing is typically subject to inversion effects in which the inverted image is harder to process/recognize. Is radiological perception similarly subject to inversion effects? Eleven experienced radiologists (>  5 years of radiological experience) and ten resident radiologists (< 5 years of radiological experience) judged upright and inverted bilateral mammograms as “normal” or “abnormal”. For comparison, the same participants judged whether upright and inverted faces were “happy” or “neutral”. We obtained the expected inversion effect for faces. Expression discrimination was superior for upright faces. For mammograms, experienced radiologists exhibited a similar inversion effect, showing higher accuracy for upright than for inverted mammograms. Less experienced radiology residents performed more poorly than experienced radiologists and demonstrated no inversion effect with mammograms. These results suggest that the ability to discriminate normal from abnormal mammograms is a form of learned, holistic processing.

## Significance statement

Breast cancer is the most prevalent cancer in women, with approximately 250,000 new cases reported each year. The ability to accurately diagnose breast cancer in an X-ray image is an essential medical skill for early detection and treatment. However, identifying breast cancer in an X-ray is not an easy perceptual task and requires many years of experience and practice. One of the key differences between an expert and novice radiologist is their ability to detect abnormalities within a medical image in the blink of an eye. It has been speculated that holistic processing (widely reported in face recognition) helps enable this rapid detection. In the present study, we test this claim by employing an inversion paradigm, which has been demonstrated to disrupt holistic processing. Resident and experienced radiologists were asked to identify abnormal and normal mammograms presented in their upright and inverted orientations. We found that experienced radiologists were more accurate at identifying abnormalities in upright mammograms than in inverted mammograms. By comparison, the radiology residents performed more poorly than the experienced radiologists overall and their performance was not affected by inversion. The expert inversion effect indicates that experienced radiologists employ holistic processes to assist their rapid detection of breast cancer and these processes are disrupted when the mammogram is turned upside down. Our results suggest that teaching methods in medical imaging may benefit by including a holistic approach in which students are trained in the rapid detection of many mammogram exemplars.

## Background

A hallmark of the human visual system is our ability to make rapid visual categorizations in fractions of a second, whether we are interpreting the meaning of a picture (Potter, Wyble, Hagmann, & McCourt, 2013), classifying a scene (Schyns & Oliva, 1994) or recognizing a familiar face (Grill-Spector & Kanwisher, 2005).

In a medical evaluation, diagnosis of chest radiographs and mammograms requires the detection and localization of the radiological abnormality (Kundel, Nodine, Conant, & Weinstein, 2007). After an initial glimpse, expert radiologists report that they have an intuition that a mammogram is likely to be normal or abnormal before any pathology is localized. In searching for signs of lung cancer, Kundel and Nodine (1975) found that radiologists could achieve a *d’* of about 1.0 after a just 200 ms glimpse of a chest X-ray. This level of performance was nowhere near the level of *d’* of 2.5 obtained during free-viewing conditions of these stimuli but, nevertheless, comfortably above chance performance. In mammography, Evans, Georgian-Smith, Tambouret, Birdwell, and Wolfe (2013) found a similar level of performance after a 250 ms exposure to mammograms. The rapid global analysis of the radiological image has been referred to as *holistic processing* and is the precursor to the subsequent stage involved in the localization of abnormalities (Carrigan et al., 2018).

In holistic processing, recognition relies on the integration of individual stimulus parts into an emergent whole representation that is qualitatively more than the summed representation of its individual parts. Face recognition is the prime example of holistic processing where recognition is based on the synthesis of facial features that yields a unique face that is more than the summed recognition of each individual facial feature. Three tasks have been applied as the gold standards for testing holistic face processes: the face composite task, the parts/wholes task, and the inversion task. In the “face composite task”, it has been demonstrated that participants find it difficult to selectively attend to one half of a face (e.g., top half) while ignoring information from the other half (e.g., bottom half) (Young, Hellawell, & Hay, 1987). In the face composite task, the whole face representation makes it difficult for participants to selectively attend to one region of the face, isolated from the whole face. In the “parts/wholes task”, participants exhibit better recognition when a face part (mouth) is displayed in the whole face than when displayed in isolation (Tanaka & Farah, 1993; reviewed in Tanaka & Simonyi, 2016). The parts/wholes task demonstrates that facial features are not represented in memory as individual parts, but are integrated into a whole face representation.

Perhaps the most widely used test of holistic face processes is the face inversion task (Yin, 1969). Although all objects are more difficult to recognize when inverted compared to upright, inversion *disproportionately* impairs the recognition of faces relative to other object classes (McKone & Yovel, 2009; Rossion, 2008; Yin, 1969). Turning a face upside down disrupts the normal holistic face processing and forces the participant to use a less optimal strategy based on analysis of specific features (wide-set eyes, square jaw, etc). Inversion has been shown to abolish the holistic interference observed in both the face composite task (Rossion & Boremanse, 2008; Young et al., 1987) and the whole face recognition advantage in the parts/whole task (Tanaka & Farah, 1993; Tanaka & Sengco, 1997).

Real world perceptual experts, such as birdwatchers or dog judges, are similar to face “experts” in that they recognize objects in their domain of expertise quickly, accurately, and at a specific level of categorization (Tanaka & Taylor, 1991). To facilitate their speeded precognition, it has been hypothesized that expert recognition demands the same kind of holistic processing that is employed in face processing. Therefore, it follows that expert object recognition should be susceptible to similar manipulations used in face recognition, such as inversion. In a seminal study, Diamond and Carey (1968) tested this prediction by asking dog judges and control participants to recognize upright and inverted photographs of dogs. They found that while the novices exhibited an inversion effect only for faces, dog experts showed a significant inversion effect for both faces and dogs. In other expert object recognition studies, inversion impairs the speed and accuracy of expert recognition processes (Ashworth, Vuong, Rossion, & Tarr, 2008; Campbell & Tanaka, 2018; Rossion & Curran, 2010; Rossion, Gauthier, Goffaux, Tarr, & Crommelinck, 2016) and limits the visual short-term memory capacity of the expert (Curby, Glazek, & Gauthier, 2009).

Although it has been speculated that mammogram expertise involves holistic strategies (Kundel et al., 2007), direct tests of holistic processing strategies in radiology have yet to be conducted. To investigate a possible link between holistic perception and mammogram expertise, we tested the effects of inversion on a group of experienced radiologists (> 5 years of radiology experience) and radiology residents (< 5 years of radiology experience). On average, an experienced mammographer evaluates between 1000 and 15,000 images per year (Evans et al., 2013) compared to resident radiologists, who see fewer than 300 cases during the course of their clinical training. Expert mammographers and residents have likely received similar formal mammography training, but it is the experts, with their extended experience, who exhibit evidence of rapid detection (Evans et al., 2013; Kundel & Nodine, 1975).

In our study, participants made a “normal/abnormal” decision to briefly presented upright and inverted mammograms. In order to rule out any age-related inversion effect, participants were asked to judge the facial expressions (e.g., neutral/happy) of briefly presented upright and inverted faces. Recognition of facial expressions, like facial identity, recruits holistic perception that is disrupted by inversion (Calder & Jansen, 2005). We made three predictions: First, given that virtually everyone is an expert in holistic expression perception, we expected that both the experienced and resident radiologists would show an inversion effect in their perception of expression (i.e., better detection of happy expressions in upright faces than inverted faces). Second, we hypothesized that the experienced radiologists (< 5 years of radiology practice) would be more accurate in their discriminations of upright mammograms than novice radiology residents. Finally, as evidence of their holistic strategies, we predicted that the experienced radiologists should show a greater inversion effect to mammograms (i.e., difference between upright and inverted recognition) than the resident radiologists.

## Methods

### Participants

Of 21 study participants, 11 were highly experienced radiologists who performed daily breast radiology screening and had at least 5 years of experience (eight female, three male; average age 56 years), average 18 years in practice (range 6 to 37 years) reading, on average, 6045 cases (range 1000 to 10,000) in the last year. The other ten participants were radiology residents who had fewer than 5 years of experience (five female, five male; average age 34 years), average 3 years in practice (range 2 to 5 years) reading, on average, 297 cases (range 20 to 500) in the last year. The expertise cut-off was based on previous studies (Evans et al., 2013; Nodine, Kundel, Mello-Thoms, et al., 1999) which suggested that radiologists with more than 5 years of experience had significantly better discrimination on rapidly presented mammograms. All study participants were recruited during the 2016 Radiology Society of North America (RSNA) conference in Chicago, Illinois, US. This study was approved by the Human Research Ethics Board at the University of Victoria, ethics protocol number 16–362. All participants had normal or corrected-to-normal vision and gave informed consent. The sample size was dictated by the availability of participants.

### Stimuli and apparatus

#### Mammograms

**Fig. 1:**
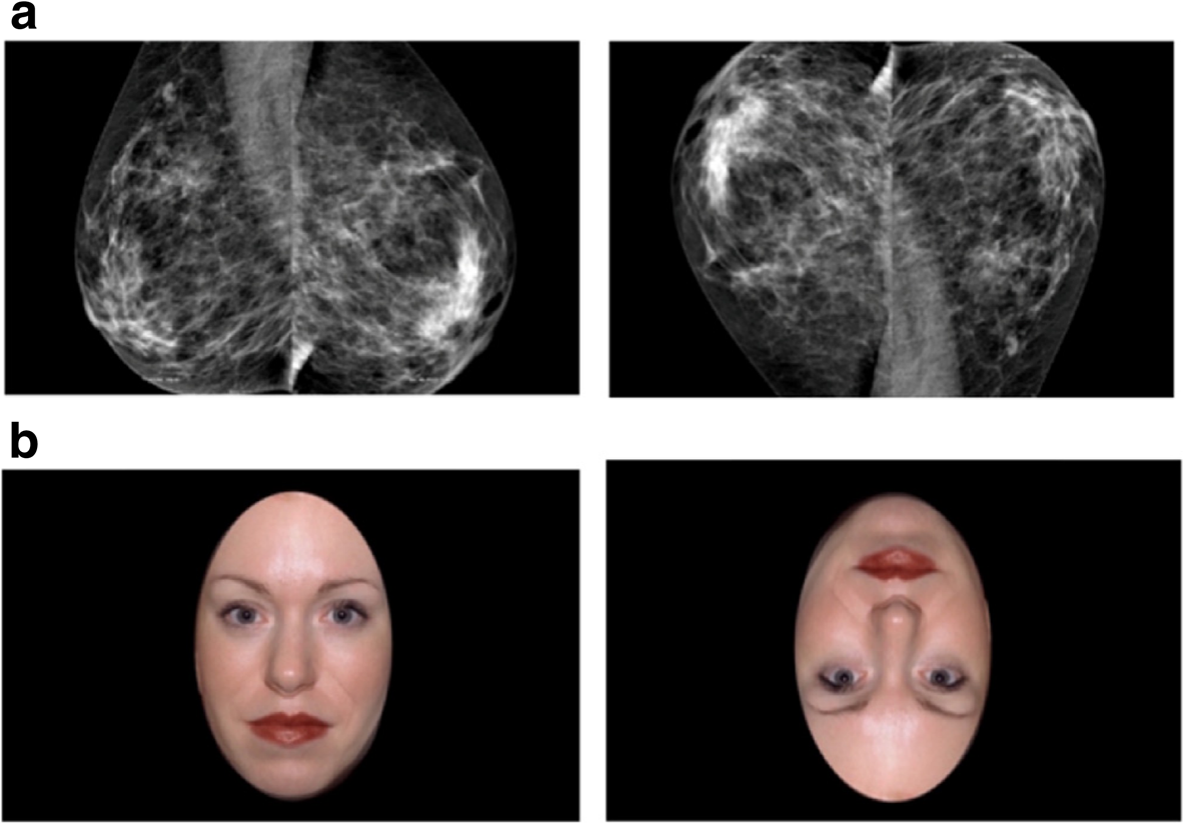
Examples of mammogram and face stimuli in upright and inverted orientations. **a** Upright and inverted abnormal mammogram. **b** Upright and inverted smiling face

Images were JPEG images of 20 bilateral full-field digital mammograms. Mammograms were presented side by side and were scaled to 800 × 500 pixels. Images subtended a visual angle of approximately 7.4° vertically and 11.9° horizontally with participants sitting 50 cm from the screen. The images showed either mediolateral oblique (MLO) views or craniocaudal (CC) views of bilateral breasts (Fig. 1a). Half of the images were normal and half showed mammograms with cancerous abnormalities. Images of abnormal cases were either histologically verified or had visible abnormalities, as determined by a study radiologist. The abnormalities were “subtle” masses and architectural distortions. Calcifications or more obvious cancers that could easily be identified by novices were not included in this study. The average size of the lesions in the test set mammograms was 18 mm (range 10–48 mm). Mammograms were obtained from anonymized cases from Brigham and Women’s Hospital, Boston, US.

#### Faces

Twenty morphs were developed from the NimStim Emotional Face Stimuli database (Tottenham et al., 2009), by overlaying a neutral and happy expression of the same individual and shifting the opacity. Faces were piloted to determine the level of difficulty that was sufficient to demonstrate an inversion effect. Higher percentage morphs (100% happy expression) were extremely salient; 40% happy, 60% neutral faces were found to be an optimal difficulty for demonstrating the face inversion effect. Control points were placed on salient features of each matching face and opacity was modified using the FantaMorphTM software package (v4.1, Abrosoft, http://www.fantamorph.com). At least 50 control points were placed on each face, and control points were added to remove obvious artifacts in the resulting morph. Hair and clothing information was removed with Adobe PhotoshopTM graphics program (v7.0, Adobe, http://www.adobe.com/photoshop). Faces were scaled to fit within a frame of 250 × 375 pixels and pasted on a black background (Fig. 1b). Images subtended a visual angle of approximately 5.6° vertically and 3.7° horizontally with participants sitting 50 cm from the screen.

The experiment was conducted on a Macintosh, MacBook Pro using in-house JavaScript scripts. All participants viewed the experiment on a 13.3-inch, liquid-crystal color screen with a 2560 × 1600 resolution, 227 pixels per inch, and refresh rate of 60 Hz.

### Procedure

**Fig. 2:**
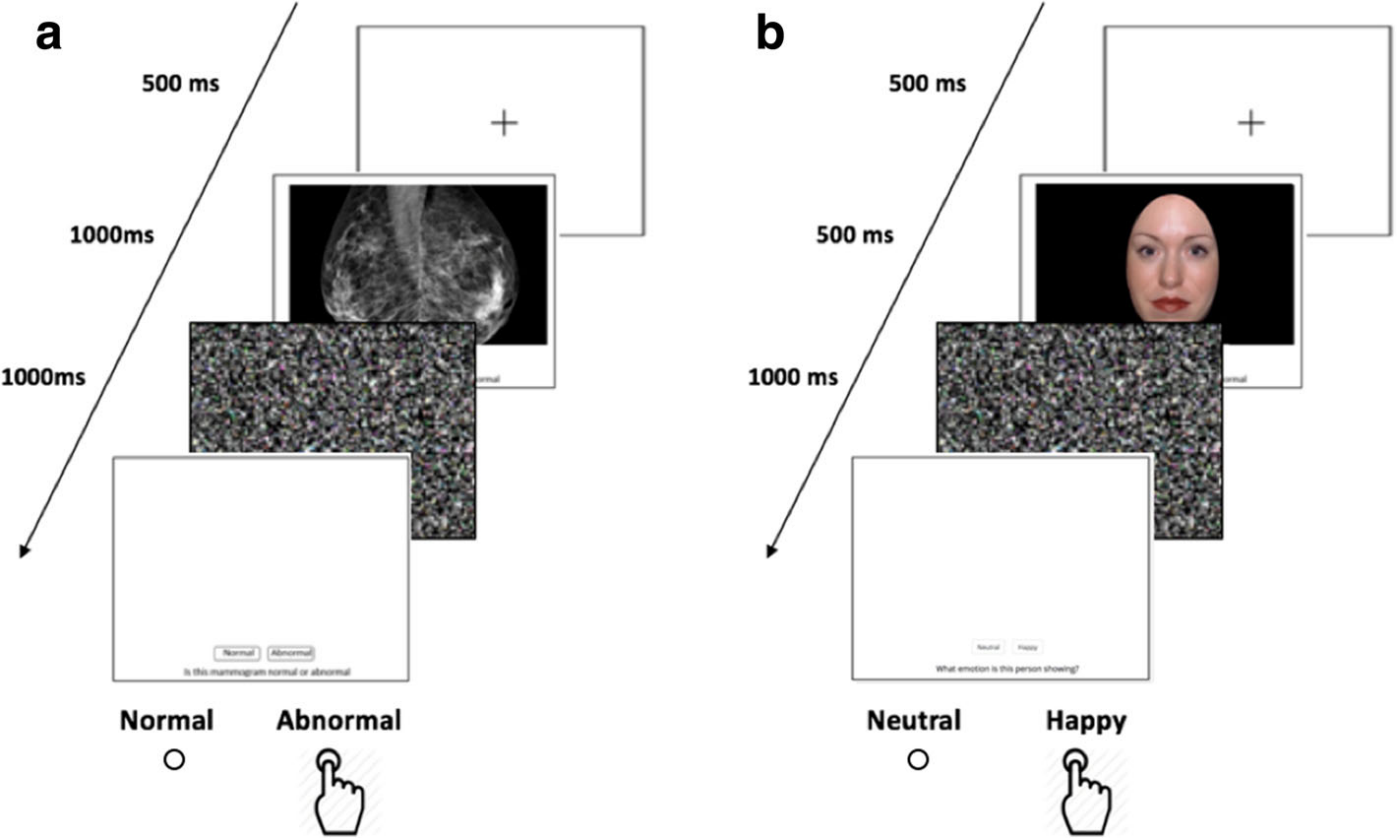
Overview of the alternative forced choice paradigm blocks. **a** Mammogram block: determine if the viewed mammogram is normal or abnormal. **b** Face block: decide whether a presented face is happy or neutral

The experiment consisted of one block of 40 face trials (ten neutral, ten happy: presented in both upright and inverted conditions) and one block of 40 mammogram trials (ten normal, ten abnormal: presented in both upright and inverted conditions). Each trial began with a fixation cross presented at the center of the screen (500 ms), followed by the target mammogram or face stimulus, a noise mask (1000 ms), followed by a response screen (Fig. 2). In the mammogram block, the participant’s task was to decide if the viewed mammogram was normal or abnormal and to indicate their response by pressing the corresponding key. In the face block, the participant’s task was to decide whether a presented face displayed a happy or neutral expression and to indicate their response by pressing the corresponding key. Face stimuli were presented for 500 ms. Though previous work shows an ability to distinguish normal from abnormal mammograms after 250 ms exposure, pilot testing in the present conditions did not produce reliable performance at that speed. Accordingly, the mammograms were shown for 1000 ms. The presentation order of the blocks (mammograms or faces) was counterbalanced across participants and participants were given a break halfway through each test.

## Results

### Faces

**Fig. 3:**
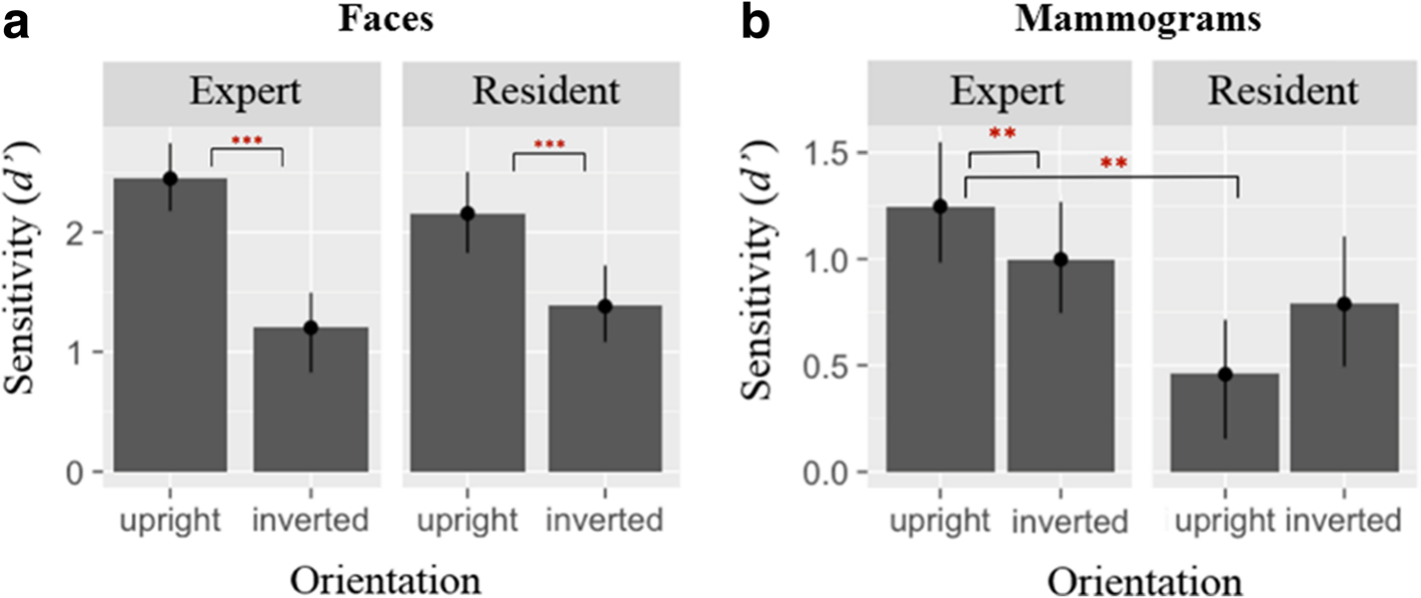
Performance *d’* scores on forced-choice task for experienced and novice radiology residents for **a** face stimuli and **b** mammogram stimuli. Error bars represent 95% CI for within-subject measures. **p < 0.01. ***p < 0.001

A 2 × 2 mixed ANOVA was conducted for the face sensitivity (*d’*), Experience (experienced radiologists, resident radiologists) was a between-group factor and Orientation (upright, inverted) was a within-group factor. A main effect of Orientation was statistically significant (F(1, 19) = 36.73, *p* < 0.0002, ηp2 = 0.53), displaying the classic face inversion effect where expression classification was impaired in the inverted orientation relative to the upright orientation. With face sensitivity, no Experience effect was found (F(1, 19) = 0.1, *p* > 0.76), nor was there an interaction between Experience and Orientation (Fig. 3a). Reaction time data for correct face trials showed a main effect of Orientation (F(1,19) = 8.49, *p* < 0.01, ηp2 = 0.06). No Experience effect was found (F(1, 19) = 0.86, *p* > 0.37, ηp2 = 0.04).

### Mammograms

For the ANOVA on the mammogram data, Experience (experienced radiologists, resident radiologists) was a between-group factor and Orientation (upright, inverted) was a within-group factor. For mammogram recognition sensitivity (*d’*), there was a main effect of Experience (F(1, 19) = 6.18, *p* < 0.02, ηp2 = 0.22; experts are better). A direct comparison using paired *t*-tests revealed that experts had better performance for upright mammograms (t(10) = 3.6, *p* = 0.0019, Cohen’s *d* = 0.45) compared to residents. There was no reliable difference for the inverted mammograms (*p* > 0.2). A Group by Orientation interaction was obtained (F(1, 19) = 11.91, *p* = 0.003, ηp2 = 0.09), reflecting the presence of an inversion effect for the experts but not residents. The inversion effect was assessed with paired *t*-tests. These reveal a significant inversion effect for experts (t(10) = − 2.8, *p* = 0.018, Cohen’s *d* = − 0.51). The effect for residents was not significant and, in any case, goes in the opposite direction from what would be expected (*p* > 0.33) (Fig. 3b). Reaction time data for correct mammogram trials showed no Experience (F(1,19) = 1.03, *p* > 0.32, ηp2 = 0.05) or Orientation (F(1,19) = 2.89, *p* > 0.11, ηp2 = 0.04) effects.

Further, we explored the differences in mammogram performance, comparing “hit” and “false alarm” rates. Experienced and resident radiologists did not differ in their ability to classify an abnormal mammogram as “abnormal” (hit rates) in upright mammograms (*t*(19) = − 0.58, *p* = 0.56, Cohen’s *d* = − 0.13). However, the experienced radiologists were less likely to misclassify a normal mammogram as abnormal (false alarms; *t*(19) = − 4.74, *p* < 0.01, Cohen’s *d* = − 1.05). This means that the criterion of experienced radiologists was more strongly biased toward a “normal” classification than less experienced radiologists (*t*(19) = 2.73, *p* = 0.008, Cohen’s *d* = 0.6). There were no significant differences in hit rates, false alarms, or biases between experts and residents for the inverted mammograms and upright and inverted faces. These results are tabulated in Table 1.

**Table 1:** Recognition performance of radiology experts and residents for upright and inverted mammogram and face trials in terms of hits (*hit*), false alarms (*fa*), sensitivity (*d’*) and bias (*c*)

Test	Group	Up (hit)	Up (fa)	Up (*d’*)	Up (c)	Inv (hit)	Inv (fa)	Inv (*d’*)	Inv (c)	Up-Inv (*d’*)
Mam	Experts	0.55	0.15	1.25	0.48	0.52	0.18	0.99	0.43	0.26 *
	Residents	0.52	0.35	0.46	0.18	0.59	0.31	0.79	0.15	−0.33
Faces	Experts	0.92	0.17	2.50	−0.18	0.63	0.19	1.36	0.26	1.19 **
	Residents	0.94	0.30	2.18	−0.50	0.69	0.24	1.33	0.15	1.16 **

*p < 0.05. **p < 0.01

**Fig. 4:**
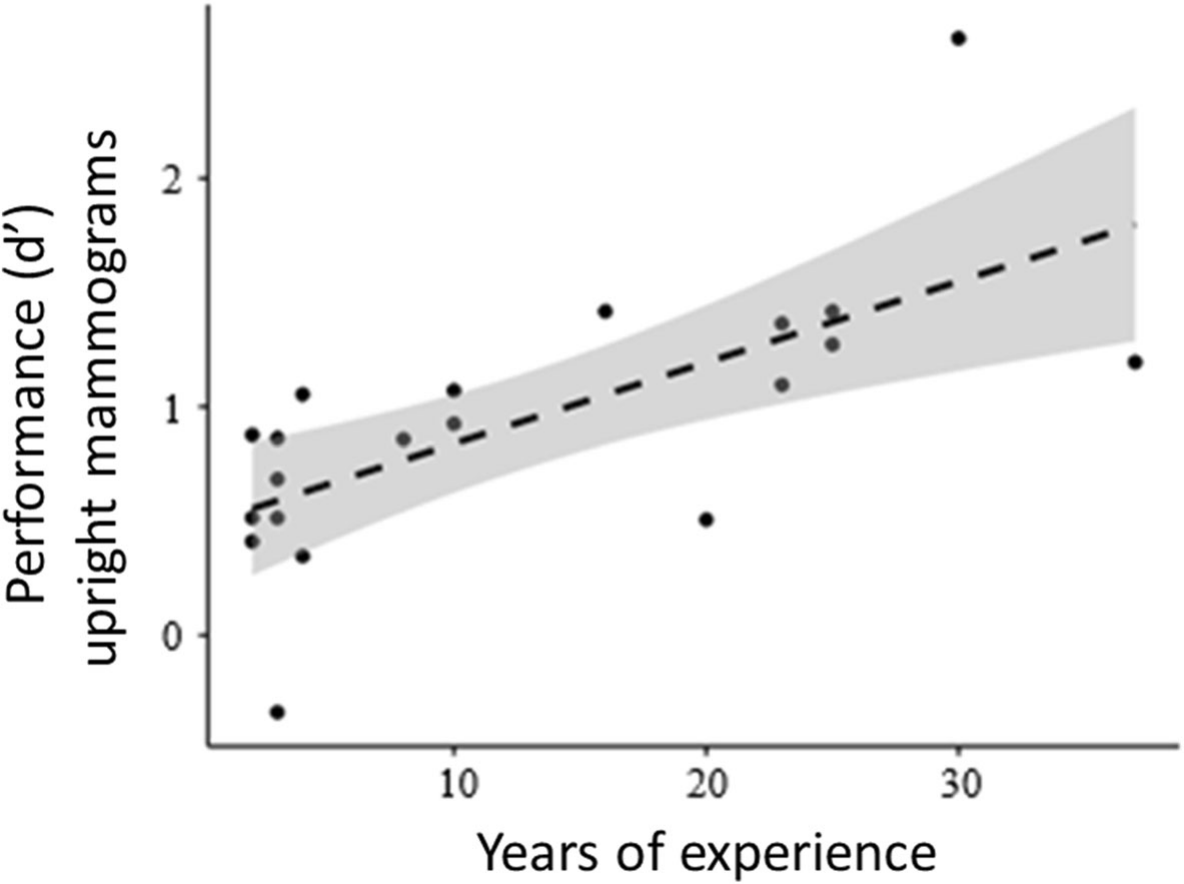
Performance *d’* scores of upright mammogram stimuli across years of radiology experience

**Fig. 5:**
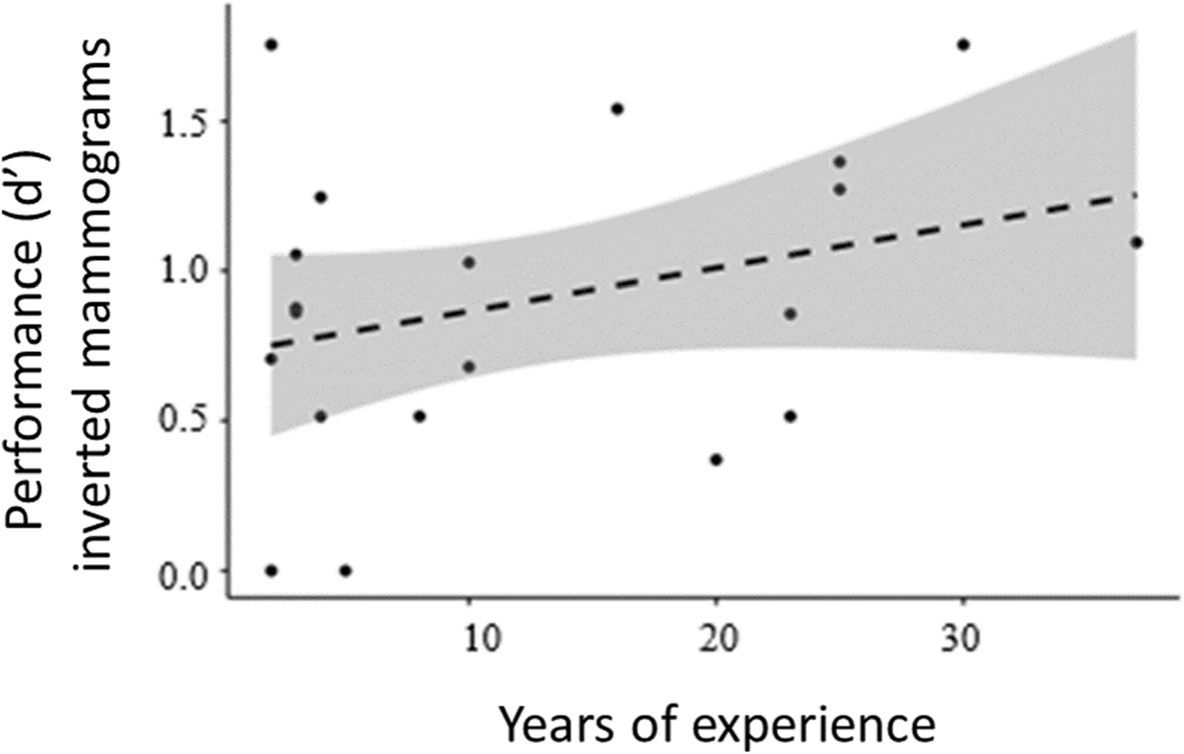
Performance *d’* scores of inverted mammogram stimuli across years of radiology experience

Figure 4 plots *d’* for upright mammograms as a function of years of experience for all 21 participants. Not unexpectedly, the results showed that mammogram discrimination improved with years of radiological experience (*F*(1, 19) = 15.2, *p* < 0.001, *R*^*2*^ = 0.45, 95% CI = 0.018, 06). However, years of experience was not related to the ability to discriminate inverted mammograms (F(1, 19) = 2.179, *p* < 0.16, *R*^*2*^ = 0.1, 95% CI = − 0.006, 0.03; Fig. 5).

**Fig. 6:**
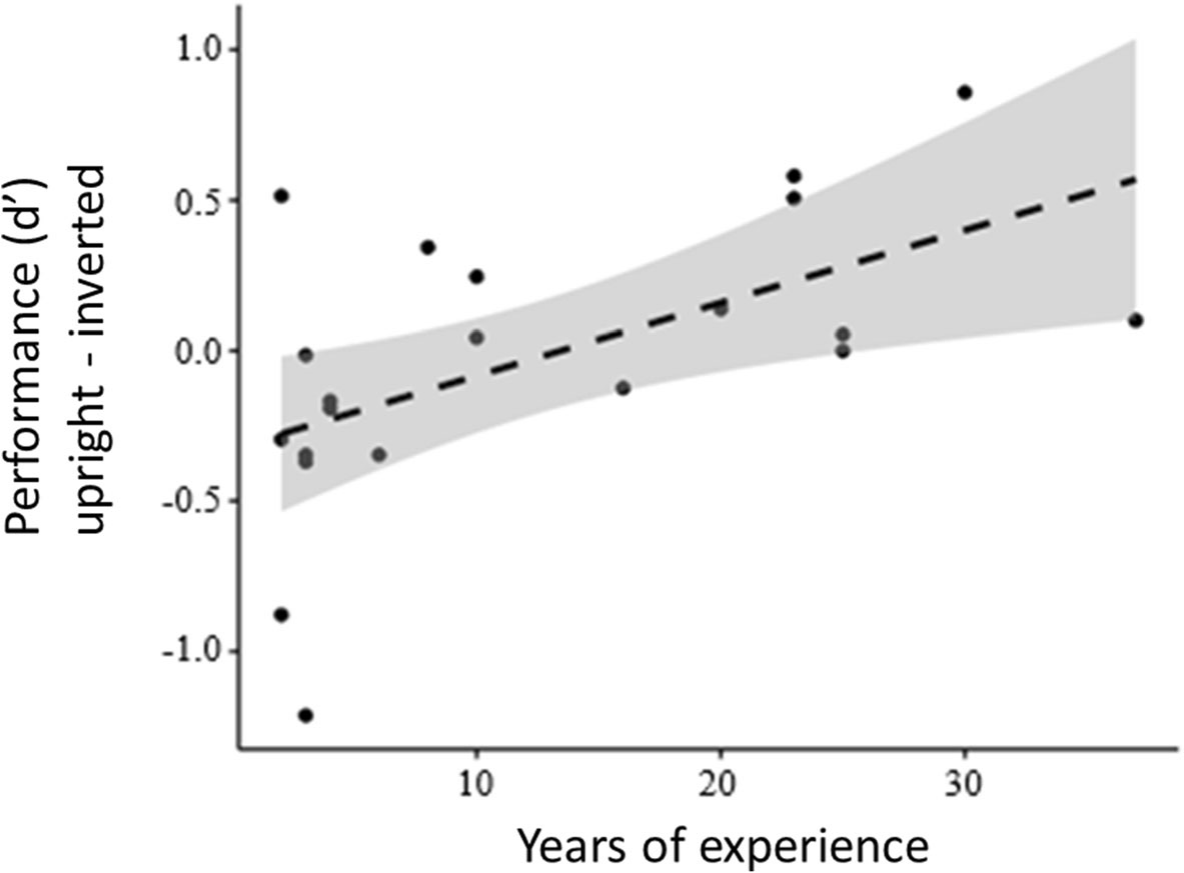
An inversion composite score of upright mammogram performance (*d’* score) minus the inverted mammogram condition performance across years of radiology experience

There is also a significant correlation of the magnitude of the inversion effect (difference between upright and inverted mammogram discrimination) with years of experience (*F*(1, 19) = 8.49, *p* < 0.009, *R*^*2*^ = .30, 95% CI = 0.018, .059; Fig. 6), suggesting that the use of holistic strategies increases as a function of radiological experience.

## Discussion

In this study, we employed the inversion task to test the holistic hypothesis of radiological expertise. Experts (> 5 years of experience) and residents (< 5 years of experience) were asked to classify upright and inverted mammograms as either normal or abnormal. As comparison stimulus, the same participants judged whether upright and inverted faces displayed a happy or neutral expression. For faces, both resident and experienced radiologists exhibited the classic face inversion effect where face expression discrimination was better in the upright orientation than the inverted orientation. For mammograms, experienced radiologists showed superior discrimination relative to resident radiologists for mammograms presented in their upright orientation. Although the overall performance of resident radiologists was worse than the experienced radiologists, their detection rates were unaffected by orientation and were essentially the same for upright and inverted mammograms. When the mammograms were inverted, the discrimination scores of the experienced radiologists dropped to the same level as demonstrated by resident radiologists. These results are consistent with previous studies of perceptual expertise showing that, with extensive domain-specific experience, experts access holistic information in an upright stimulus, but holistic information is impaired when the stimulus is inverted (Campbell & Tanaka, 2018; Diamond & Carey, 1986; Rossion, Gauthier, Goffaux, Tarr, & Crommelinck, 2016).

Although inversion impaired mammogram detection for the experienced radiologists, it did not completely abolish the expertise advantage. The performance of experienced radiologists in this study (*d’* = 0.99) was comparable to detection levels of expert radiologists in previous studies for upright mammograms (*d’* = 1.14) (experiment 1; Evans, Haygood, Cooper, Culpan, & Wolfe, 2016), albeit at a shorter exposure presentation of 500 ms. The residual expert effect for inverted mammograms suggest that other types of non-holistic information survived the inversion manipulation. Harley et al. (2009), for example, presented chest radiographs to experts for 500 ms and compared normal images to chest radiographs that were scrambled (e.g., segmenting image into 25 squares and shuffling their positions). Despite disrupting the global structure of the stimulus, the experts’ performance dropped slightly from *d’* = 1.23 for the intact stimulus compared to *d’* = 1.09 for the scrambled stimulus.

The holistic account of mammogram expertise is consistent with eye-tracking studies which show that, in comparison to non-experts, experts typically perform domain-related tasks with fewer fixations, longer saccades, and less coverage of the image (Krupinski, 1996; Manning, Ethell, Donovan, & Crawford, 2006). One study directly examined the eye position of expert breast radiologists and of novice radiology residents when reading digital mammograms (Kundel et al., 2007). They found that the median time for the eyes of the experts to reach the location of a cancerous nodule was 0.96 s from image onset whereas for the novices that time was 2.15 s. The authors speculated that response time for the experts was too fast to support a search-to-find strategy, implying that their search was being guided by a global representation of the mammogram. In the current study, the exposure duration was increased to 1000 ms in order for the experienced and resident participants to perform above chance levels. The extended exposure duration contrasts with previous “gist” studies where expert radiologists achieved reasonable detection scores after a much shorter presentation duration (i.e., 250 ms or less; Carrigan, Wardle, & Rich, 2018; Evans et al., 2016; Kundel & Nodine, 1975). Given that inverted and upright mammograms were randomly presented in the current study, it is possible that additional encoding time was required to, first, determine the orientation of the stimulus and, next, to apply the appropriate holistic and non-holistic strategy. In future studies, it would be useful to block the mammogram stimuli by orientation so participants will have the opportunity to prepare their detection strategy prior to the onset of the stimulus.

The emergence of a holistic strategy with radiological experience has implications for training medical students. In our study, the expert radiologists acquired holistic strategies implicitly over many years of clinical experience, evaluating thousands, even tens of thousands of mammograms images. It is interesting to speculate whether this perceptual knowledge can be taught explicitly during medical training by presenting medical students with many images of abnormal and normal mammograms, asking them to judge the normality of each image and providing the appropriate feedback. A perceptual expertise protocol might accelerate the learning process and allow radiological trainees to achieve expert performance more quickly. The perceptual expertise training approach has been successfully applied in other medical domains that require visual diagnosis. For example, participants showed reliable gains in their ability to detect melanoma skin lesions after four 30-min sessions of perceptual expertise training (Xu, Rourke, Robinson, & Tanaka, 2016).

## Conclusions

The goal of this study was to measure the holistic processing of expert mammographers by employing the inversion test—a standard measure of holistic processing used in the face recognition research. The main finding was that experienced radiologists exhibited a robust inversion effect as evidenced by their better discrimination of upright mammograms than inverted mammograms. In contrast, the less experienced, resident radiologists performed more poorly than experienced radiologists and their discriminations were the same for upright and inverted mammograms. Critically, detection performance improved and the inversion effect increased for radiologists who had more experience diagnosing mammogram images, suggesting that holistic abilities developed as a function of perceptual experience. Our results have implications for training medical students by emphasizing the role of experience and the learning principles of perceptual expertise.
